# Supplementary material for: Parvovirus B19 and Human Parvovirus 4 Encode Similar Proteins in a Reading Frame Overlapping the VP1 Capsid Gene
Source: Viruses. 2024 Jan 26;16(2):191. doi: 10.3390/v16020191 (PMC10891878; doi:10.3390/v16020191)

S3

A. Splicing profile of Parvovirus B19

P6: promoter

D1, D2: splicing donor sites

A1-1, A1-2, A2-1, A2-2: splicing acceptor sites

pAp1, pAp: proximal poly-adenylation sites

pAd: distal poly-adenylation site

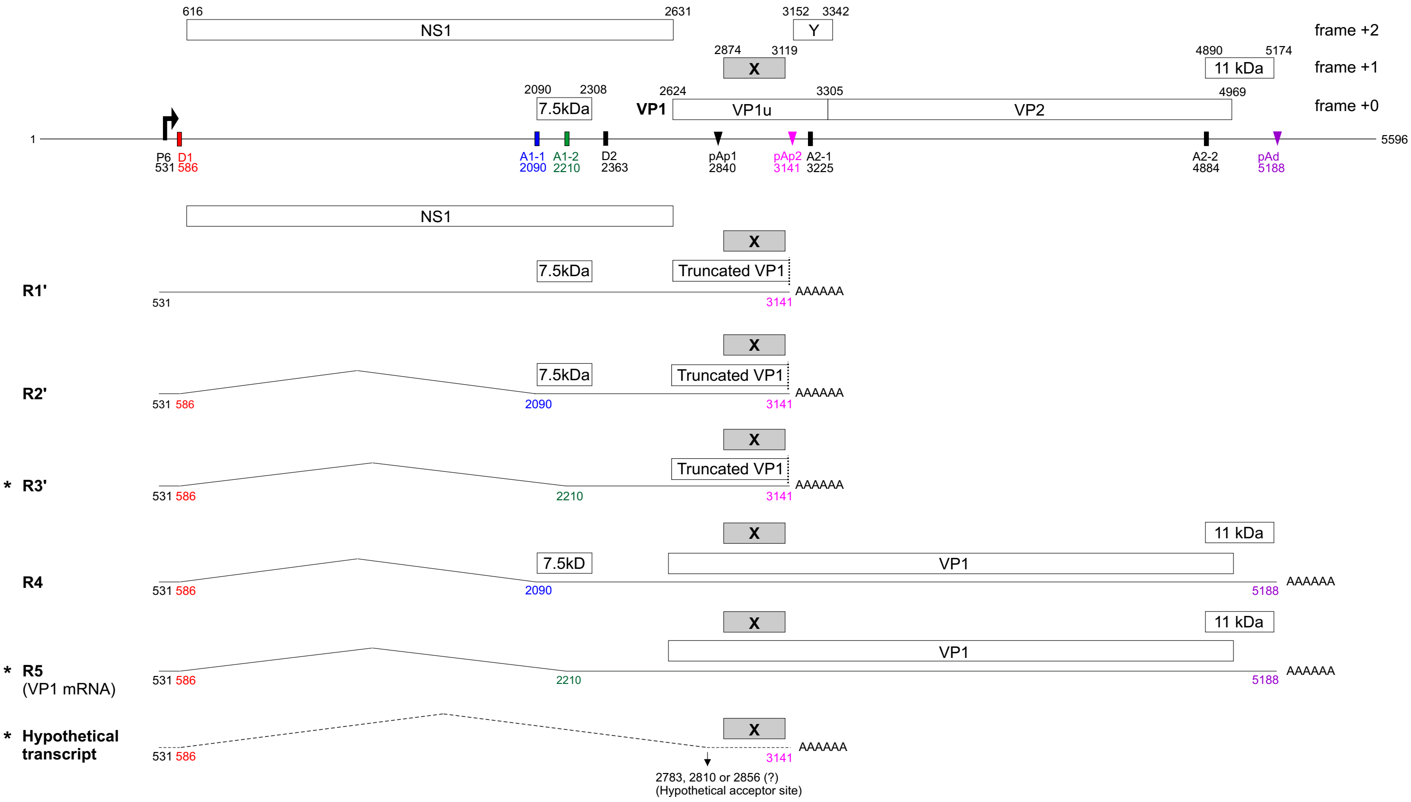

B. Splicing profile of Human parvovirus 4

P6, P38: promoters

D1, D2, D3, D3m: splicing donor sites

A1, A2, A3: splicing acceptor sites

pA: poly-adenylation site

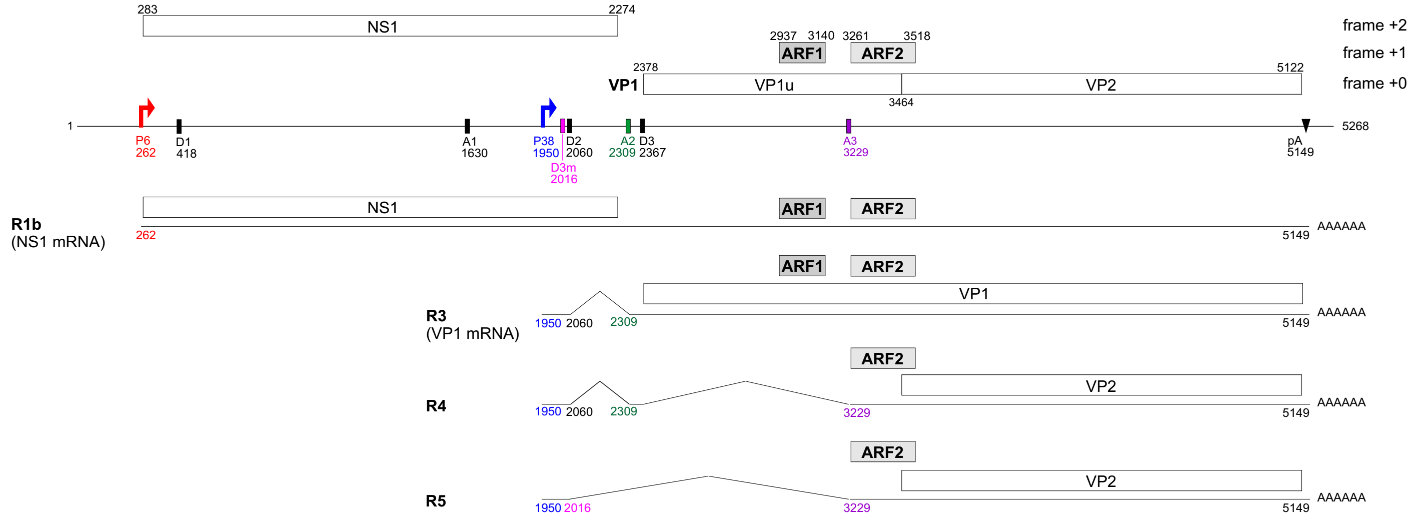

Supplement: Supplementary file 1 [file viruses-16-00191-s001.zip › S3_Fig Splicing pattern parvovirus B19 and parvovirus 4.pdf]
