# Supplementary material for: Parvovirus B19 and Human Parvovirus 4 Encode Similar Proteins in a Reading Frame Overlapping the VP1 Capsid Gene
Source: Viruses. 2024 Jan 26;16(2):191. doi: 10.3390/v16020191 (PMC10891878; doi:10.3390/v16020191)
Supplement: Supplementary file 1 [file viruses-16-00191-s001.zip › S5 Alignment_start codons of X protein.pdf]

S5 Alignment. The X and ARF1 ORFs have a potential AUG start codon in all erythro- and tetraparvoviruses, respectively

Codon-based nucleotide alignment of the region surrounding the start of the X ORF of erythroparvoviruses (panel A) and of the ARF1 ORF of tetraparvoviruses (panel B). These alignments are derived from the reference alignment of the VP1 protein using TranslatorX. Numbering corresponds to the VP1 CDS.

Putative AUG start codons are highlighted in yellow. For each species, the putative AUG start codon is conserved in all isolates (not shown in this alignment). Note how the AUGs tend to be in a similar location within erythroparvoviruses (except in chipmunk parvovirus and seal parvovirus), and within tetraparvoviruses.

UAG or **UGA**: stop codon

AUG: putative start codon of the X ORF

A. Erythroparvoviruses

```
Parvovirus B19       185 UAGUUGCUCGCAUUAAAAAUAACCUUAAAAAUUCUCCAGACUUAUAUAGUCAUCAUUUUCAAAGUCAUGGACAGUUAUCUGACCACCCCCAU---------GCCUUAUCAUCCAGUAGCAGUCAUG 301
Simian parvovirus    293 ACCUUAAA---CAACAUUUACAAGACUAUAUAGACAAUCCAGAUAAGUACACUUUAGACUUGUCUCAUGGACCUCUCCCAGAUUUCAGAGAA---------ACUGAGGCAGAACACAAAUCCUUUA 406
Rhesus macaque       299 AUCUACAA---AAACAAAUUGAAAACUAUAAAAAUAAUCCAAACAAAUAUACAUUACAGUUGUCUCAUGGACCUCUCCCUGAUUUCAGAGAG---------UCUGAGGCAAAACAUGAAUCCUCUA 412
Pigtailed macaque    194 UGGUUAAU---CGCAUUAAAAAAAAAUUUAAAAGACAAUCCUGAUAUUUACACUGACUCCUUAAGUCAUGGCGCUCUCCCAGAUUUCCGAGAA---------UCUAAGGCUGAGCAUGAGAAGUCUA 307
Chipmunk parvovirus  311 CCGUAGCU---AACACAGCCAAGCGGUUAAAAACUGACGAGGAUCCCUUA------UCCUUUGGGGCCCCCCACUAACAGAAAACGCCCCGGUUCCCGUUGCGGAGCCAGAUGUGGCAAUUGUUU 427
Seal parvovirus      338 GGAGGCACUAUGGAUCCUCAAAGGAGCAGGGCGCAGCACCCCCCGCUGAUCCCAGACAUAUAGCAGGCCCUCCUCUCACAUCACAGGGCCCG---------GUGUUGGGGGAGCAUGUCCCUAUCA 454
```

B. Tetraparvoviruses

```
Human parvovirus 4   467 UUUUUGAA---GACUCGCUAACGAACUUUGCCAAAGAAGACUUAGACACCUGGCAACAACUCCACGAGCAGUUUAUCAAACUCUUUCACCCU---------CCAGAUGUCGGAGUCCACCUUGUUA 580
Porcine hokovirus    464 UCUUUGAG---GAGAGUGUAUCUCCUUGGUCUGAGGAAGACAAAAAAAUUUUGAAACAGAUUGAAGGGCAGUUCCAGGAAAUAUUCCAUCCACCCACAGAUACGGAGGAUGGAGCCGAUAGCUACG 586
Yak hokovirus        491 UUUUUCAG---GGCAUGUUAUCUCCCAUCAAACCUGAAGAUAGACCUAUAUUAGACACCAUACAGAAGCAGUUUGAGGAGUUUUUUUAUCCUCCUAUAGUGGAACAUGCUGGACCAGGCGACUACA 613
Deer tetraparvovirus 491 ACUUUGAU---GGCAUGUUGUCACCUAUUCCAGUGGAACAACAUCCAAUUGUAGAGCAGAUAAAGGCUCAGUUUUUAAAUAUUUUUCAUCCGCCACCUAUAGCUGGAGAUGGAGUCGGUGGUGACG 613
Ovine hokovirus      491 UUUUCAAU---GGUAUGUUGACGCCGUUUGAUUCCGACCAGCGACCGGUGGUCGAGCAGAUUCACCAGCAGUUCUUAGACAUCUUUCACCCCCCACCAGUGCAUGGCCAUGGCGGAGGUGUCGACA 613
Rodent tetraparvo    467 ACUUUGAC---CAGUUCAUCACCCCGGUGGACCCCGGGGAGCCCGAGAUUCUAAAGCAGGCCUUCCAGCGCAUGGUAGAAUAUUACCACCCCGCCCCU---CAGGAUGGCGGACCUGCUCCUCCCC 586
Eidolon parvovirus   458 UUUUUGAU---GAGUCUGUGCUGGGCAGCGUUAGCGGAGACCCCGAAAACUUUGCUUUUGUGAAGCAGGCAGUAGUUAGUGCUUUUCAUCCUGCA------GAUGGAGGAGCCGCUACUGAACAGC 574
Oppossum tetraparvo  440 UUUUUACU---GACUCUAUUAGUGCGGGUGUAGCUGGAACA---CCACUCUGGGGAGAUACUGCAGAAGCUCUCAUGUCGGCCCUU---------------GCAGGGAAUGGAAGUAUCAGAGGGU 544
```
