# Supplementary material for: Parvovirus B19 and Human Parvovirus 4 Encode Similar Proteins in a Reading Frame Overlapping the VP1 Capsid Gene
Source: Viruses. 2024 Jan 26;16(2):191. doi: 10.3390/v16020191 (PMC10891878; doi:10.3390/v16020191)
Supplement: Supplementary file 1 [file viruses-16-00191-s001.zip › S6 Alignment_X erythro and tetraparvoviruses including Indri and hyaena endogenous parvoviruses.pdf]

S6 Alignment. Alignment of the X protein of erythroparvoviruses (including reported endogenous erythroparvoviruses) with the ARF1 protein of tetraparvoviruses.

The alignment, in FASTA format, includes two sequences reported to come from endogenous viruses: that of Indri indri endogenous parvovirus and Hyaena hyaena endogenous parvovirus [1].

```
>ParvovirusB19/1-81
------------------------------------MDSYLTT-PMPYHPVAVMQNLEEKMQYYLVKTYTSL
GKLAYNYPVLTMLGLAMSYKLGPRKVLLTVLQGFMTLGIANWLSWE--------------------------
--------
>SimianParvo/1-81
------------------------------------MDLSQIS-EKLRQNTNPLMNLEEMTQSLLKKTYTKG
VELVLHYPLVTTLALVISFKLGIPKVWWTLRLGFMILGILNLLNWE--------------------------
--------
>RhesusMacaque/1-79
------------------------------------MDLSLIS-ESLRQNMNPLMNLEEMTQSLVKRIYTKG
VDLVCNYPSVITLALVMSYRLGPRRVLWMLQLGVMISGILNLLS----------------------------
--------
>PigtailedMacaque/1-79
------------------------------------MALSQIS-ENLRLSMRSLMNLEELTQSLLVKTYTMG
VQLVSHYPSLTILGLGILYKLGPQRTWWMLQLGFTITGILNLLS----------------------------
--------
>IndriEndogenousParvovirus|RJWJ010000280/247-327
------------------------------------MNLTLVN-HLLNELNLTPPLLEQFLRDSGKTLLTYL
TEVVYTYPATATLVLATLWKTGLPLIWWTRPPGSTTSGTQKSKQKA--------------------------
--------
>HyaenaParvovirus-like|PITC01058066.1_X protein Hyaena hyaena, whole
genome shotgun sequence 2000-3406/223->1405
MRGVIISQFQSTLLNQSLLLISAPPRSQRLGLRWRLMSLAPPS-TWRET----ILCLENSLQRAISE-----
---VSHFPAVTTLGLAILWIQGPRLLSWTQRLAYTILGIHNYKAWGWILTPTLLRPTKSCCQPRLEKRDFRP
PW------
>SealParvo/1-103
------------------------------------MSLSQNR-SWQEI----LSPLESGLQTMVIE-----
---ALHYPALTTLGLAIAWKKGPQLLGWTLRLAYTILGIVSSRSWALIPTHTSHPLTRNSSRQPPEKRVSRQ
QWSRLSLS
>ChipmunkParvo/1-65
--------------------------------MW-QLFLNQTE-TLLQN------NLNEAWQSLITGAST--
------YPLTGILALAILWKTAPLLTRLTQWLVFTTLGTLT-------------------------------
--------
>Human_parvovirus_4/1-83
----------------------------------MSESTLLVT-AAMKE---LIPLLNQTLSG-LPEA----
---GLLSPGIIMLVLVILWIVVPLRDQWMRQQNIMMNGTQR-LSMGTSLIYMVTALTD--------------
--------
>PorcineHokoHK7/1-84
-----------------------------------MEPIATVA-TVIKE---LVPLVNQTLNA-LPSAP---
---ESWSPGTIMLVLVILWIMLPLRDQWMKQRNTTMNGTMKCFAMVICHTSMGEGLIG--------------
--------
>YakHoko/1-84
----------------------------------MLDQATTVA-TVIRE---LVPLVNQTLSA-LPTA----
---DSWSPATIMLVLVILWIMVPLRDQWMRQRSTTMNGTMKCCLMATCHMFMVTGQID--------------
--------
>OvineHoko1/1-85
---------------------------------MAMAEVSTLA-TVVQE---LLPLVNQTLND-LPKG----
---DFWSPVTIMLVLVILWIMVPLRDQWTRQRNIMTNGTMKCYHMGMSHIYTDTGLTD--------------
--------
>DeerTetraparvo|NC_031670_1_cds_YP_009315887_1_2/1-/1-84
-----------------------------------MESVVTVA-TVLEE---LIPLVNQTLNA-LPKG----
```

```
---DFLSPVIVMLVLVILWIMAPLRAQWTRPLNTMTNGTQICYLMGTYHTSMVMEQTDL-------------
--------
>RodentTetraparvo/1-85
----------------------------------MADLLLPLS-SIMRE---LLPALNHTATE-LAREG---
---VSWSPGTIMLVLVILWIMAPLRDRLMRPQRDTMNGTMRCSSMGTCPICMAMGRMA--------------
--------
>EidolonParvo1/1-77
----------------------------------MEEPLLNSALTSLKV---LAPLVNQTIHS-LAEV----
---VSLSPATIMWVLVILWIMAPLRAQWMRQLAIMTEGMTKCFPTGTSPT----------------------
--------
>OppossumTetraparvo/1-76
--------------------------------MEVSEGLQLLR-TIAQ------ATANQTAEAILPGA----
---GSLSPAIIMLVLVILWIMAPLRAQWMRRLNAMTNGMMSCWVMGMCHT----------------------
--------
```

References

1. Campbell MA, Loncar S, Kotin RM, Gifford RJ. Comparative analysis reveals the long-term coevolutionary history of parvoviruses and vertebrates. Quental TB, editor. PLOS Biol. 2022;20: e3001867. doi:10.1371/journal.pbio.3001867
