# Supplementary material for: Parvovirus B19 and Human Parvovirus 4 Encode Similar Proteins in a Reading Frame Overlapping the VP1 Capsid Gene
Source: Viruses. 2024 Jan 26;16(2):191. doi: 10.3390/v16020191 (PMC10891878; doi:10.3390/v16020191)
Supplement: Supplementary file 1 [file viruses-16-00191-s001.zip › S7 Alignment_PLA2 domain of erythro, tetra, and related parvoviruses for phylogenetic analysis.pdf]

Alignment S7: Sequence alignment of the PLA2 domain of representative erythro-, tetra-, copiparvo-, bocaparvo-, dependoparvo-, and protoparvoviruses.

This alignment was used for the phylogenetic analysis of Fig 9.

```
>B19|YP_004928146.1/123-182 [Human parvovirus B19]
VQLPGTNYVGPGNELQAGPPQSAVDSAARIHDFRYSQLAKLGINPY-THWTVADEELLKNI
> Primate Erythroparvovirus 4|AAF61214.1/125-184 capsid protein [Pig-tailed
macaque parvovirus]
LTLPLTHYIGPGNPLQAGSPTDVVDAAARIHDYRYSELIKLGINPY-THWTVADDELLHNV
>Primate Erythroparvovirus 2|AAA74974.1/158-217 VP1 [Simian parvovirus]
LTLPFSNYIGPGNQLQAGNPQSVVDAAARIHDFRYSELIKLGINPY-THWSVADDELLHNI
>PrimateErythro3|AAF61211.1/160-219 capsid protein [Rhesus macaque parvovirus]
VQLPFSHYIGPGNELQAGAPESVVDAAARSHDFRYSELIKLGINPY-TQWTVADDELLHNI
>Seal parvovirus|AHA86836.1/175-234 VP1 [Seal parvovirus]
LTLPGTHYVGPGNRLEEGPPTTRLDAEARIHDFRYSELQKLGINPY-THFTSADQELLSAA
>Chipmunk parvovirus|ACT09662.1/166-225 VP1 [Chipmunk parvovirus]
IHLPADRYLGPGNPLENGPPVDPVDAVARIHDFRYADLEKQGINPY-TTYTIADEELLKNL
>Bocaparvovirus primate1|YP_338088.1/11-70 VP1 [Bocaparvovirus primate1]
WVLPGYRYLGPFNPLDNGEPVNNADRAAQLHDHAYSELIKSGKNPY-LYFNKADEKFIDDL
>Rhinolophus Bocaparvovirus 2|YP_009553049.1/11-70 VP1/VP2 [Rhinolophus
pusillus bocaparvovirus 2]
WVVPGYKYLGPFNQLDAGEPVNKSDQAAQKHDFAYDQYIKSGKNPY-LYFNKADQDFIEDL
>Canine minute virus|NP_758523.1/11-70 virus protein 1 [Canine minute virus]
WVVPGYKYLGPFNPLDNGTPINKVDKAAQKHDFAYQSYINKGENPY-LNFNKADSDFIEDL
>Feline Bocaparvovirus 2|YP_008802582.1/11-70 VP1 [Feline bocaparvovirus 2]
WTVPGFKYLGPFNPLDNGDPVNEVDKTAQTHDRAYQSYIDAGINPY-LNFNKADSDFIESL
>Dromedary bocaparvovirus 1|YP_009389294.1/13-72 VP1 [Dromedary camel
bocaparvovirus 1]
ILFPGYNYLGPFNPLDNGEPVNKADKAAKRHDLAYNQYLNKGLNPY-LKFNKADQQLIDDL
>Canine bocavirus 3|YP_010802373.1/11-70 VP1 [Canine bocavirus 3]
WLFPGYKYLGPFNPLDNGEPVNKADSVAREHDLAYSKYLEAGKNPY-LNFNKADQKFIDDL
>Rat bocavirus|YP_009227293.1/11-70 VP1 [Rat bocavirus]
WVLPGFKYLGPFNPIRSGKAKNRVDQAALRHDYAYDRYINNKVNPY-FKFNKADQTFLNDL
>AAV2|YP_680426.1/45-104
LVLPGYKYLGPFNGLDKGEPVNEADAAALEHDKAYDRQLDSGDNPY-LKYNHADAEFQERL
>AAV12|DQ813647.1_cds_ABI16639.1_2/45-104
LVLPGYKYLGPFNGLDKGEPVNEADAAALEHDKAYDKQLEQGDNPY-LKYNHADAEFQQRL
>Bearded dragon parvovirus| YP_009154713/40-99
LVVPGYKYLGPFNGLDKGEPVNAADAAALEHDKAYNELLEAGDNPY-IKYNHADAVFQERL
>Rhinopholus sinicus AAV1|MF682927/48-107
LVLPGYKYLGPFNGLDKGQPVNKADEVAREHDLEYNKLLEAGDNPY-LKYNHADQEFQEKL
>AAVpo7|KM349848.1_cds_AIU56932.1_1/44-103 [porcine AAV 7]
LVLPGYNYLGPFNGLDKGEPVNRADAVAREHDISYNEQLQAGDNPY-LKYNHADAEFQEKL
>Bovine AAV|YP_024971/44-103
LVLPGYKYLGPGNGLDKGDPVNFADEVAREHDLSYQKQLEAGDNPY-LKYNHADAEFQEKL
>California sea lion AAV1| YP_009507367/48-107
LVLPGYKYLGPFNGLERGEPVNAADAAAQRHDRQYDRILQQGGNPY-LTYNHADREFQEEL
>Goose parvovirus|NP_043515/53-112
FVLPGYKYLGPGNGLDKGPPVNKADSVALEHDKAYDQQLKAGDNPY-IKFNHADQDFIDSL
>Snake AAV| YP_068094/44-103
FVVPGYRYLGPGNSLDRGKPVNKADEAAKKHDQEYDQQLKAGDNPY-IKYNHADEQFQKDL
>Slow loris parvovirus1|YP_009111340.1/110-169 VP1 [Slow loris parvovirus 1]
FVVPGYKYMGPGNDPDVGPPVNAADTAARDHDLRYAQMQNAGINPY-VKYNKADEKMIEEL
```

>DesmodusRotundus|MG745677/33-92
WVWPGYKYLGPGNSLDKGEPVNALDAAARLHDIGYSRLQEKGVNPY-LLYSSVDADFQSAI
>OppossumTetra|AVR53745.1/206-265 capsid protein 1 [Opossum tetraparvovirus]
LTLPGYNYVGPGNPLDNGPPQGPVDEAAQRHDQRYDELLGHGDVPY-LNSHGADQMMSEEL
>Eidolon parvovirus 1|YP_005090505.1/215-274 unnamed protein product [Eidolon helvum (bat) parvovirus]
LLVPGYNYVGPGNPLDNGPPKGPVDEAARNHDRRYDEMLSHGDVPY-LDSQGVDQLMTKEI
>RodentTetra|AVR53743.1/220-279 capsid protein 1 [Rodent tetraparvovirus]
LVVPGYNYVGPGNPLDNGPPQGPVDEAAKRHDERYDEMLKHGDMPY-LHGHGADGMMGKEI
>Ungulate tetraparvovirus 1|YP_009175069.1/228-287 minor structural protein [Ungulate tetraparvovirus 1]
LLVPGYNYVGPGNPLDNGPPQGPVDEAAKHHDERYDEMLSHGDLPY-VHGHGADRLMNKEI
>Unclassified tetraparvovirus|YP_009315887.1/228-287 VP1 [Tetraparvovirus sp.]
FLVPGYRYVGPGNPLDNGPPEGPVDEAAKHHDERYADMLSHGDVPY-LHGHGADRLMNREI
>Human parvovirus 4|YP_238483.1/217-276 ORF2 [Human parvovirus 4 G1]
LTLPGYNYVGPGNPLDSGPPQGPVDEAAKHHDERYAEMIEHGDIPY-LHGHGADRLMNKEL
>Ungulate tetraparvovirus 2|ABY67707.1/220-279 minor structural protein [Porcine hokovirus]
ILVPGYNYVGPGNPLDNAPAKGPVDEAAKHHDERYDEMLRHGDLPY-IHGRGADRLMNKEI
>Ungulate tetraparvovirus 3_PorcineParvo2|YP_009389279.1/278-337 gp2 protein [Ungulate tetraparvovirus 3]
LTLPGYRYVGPGNPLDAGEPRGPVDAIAKKHDERYDELIKHGHIPY-IHGRGADTMMGKEL
>Copiparvovirus pangolin|URG17256.1/307-366 capsid protein [Copiparvovirus P229T/pangolin/2018]
LVYPGHKYTGPGNPQDSGPPTGPIDESSQKHDLRYGALLDHGELPY-ICAKEVDKLQSRDI
>Sesavirus|YP_009116877.1/233-292 VP1 [Sesavirus CSL10538]
LTVPGTSYVGPGNPVPAGPPSGPVDQAALEHDQRYSWLLSLGDWPY-LLANEADKIMSDDL
>Equine parvovirus H|AUW64491.1/200-259 virion protein [Equine parvovirus H]
LLFPGTHYLGPGNPVPNGPPVDKLDAAAAKHDIRYDAMLKHGDIPY-LKHNWADEAMSRDL
>Porcine parvovirus 6|YP_010796330.1/414-473 capsid protein [Porcine parvovirus 6]
FVLPSHHYTGPRNPVPAGKPADPVDESSARHDIRYGQRLKHGDWPY-LWGKDLDNAQRDEI
>Porcine parvovirus 5|YP_008888534.1/245-304 capsid [Porcine parvovirus 5]
INWPGHRYTGPGNPLPHGAPRNEIDLSAAKHDIRYKQYSRYGHWPY-IWAPYIDKKMQEDI
>Ovine copiparvovirus|QYW06846.1/131-190 MAG: capsid protein [Ovine copiparvovirus]
LVWPGYKYLGPGNDLQQGPPVDELDAAAMRHDVRYATFLSEGDIPY-IWAPEIDKQMIKEI
>Copiparvovirus ungulate5|QVK82310.1/296-354 VP1 [Copiparvovirus ungulate5]
LTYPGTRYVGPGNLVPAGPPVNPIDDLALQHDLRYGTVMEHGEWPY-IY-DESDDILAQDI
>SikaDeer copiparvovirus|BDE68838.1/316-374 structural protein [Sika deer copiparvovirus]
LTYPGSRYIGPGNLIPYGPPTDDIDNAAALHDIRYETMISHLHWPY-IY-DQSDDYLIEDI
>Bovine parvovirus 2|APC23635.1/92-151 structural protein [Bovine parvovirus - 2]
LTLPKHRYVGPGGDLPAGRPMSKLDEIAARHDIGYHTEITHKHNPY-YWYNFYDEQMVKEI
>Bovine copiparvovirus 3|UQW95854.1/278-337 structural protein [Bovine copiparvovirus 3]
IGIPGTHYIGPGSKVPGPKPTSKLDEIALHHDLGYEQLLAHGEWPY-INYNYYDEKMIQEI
>Horse parvovirus CSF|AKN50610.1/351-411 capsid VP1 [Horse parvovirus CSF]
WTWPGKKYCGPGNKVPCGPPKDKVDECSALHDIGYEKLIKEGKWPYGCEGCGADSKMIECL
>Canine protoparvovirus|YP_010797498.1/11-70 VP1 [Canine protoparvovirus]
WVPPGYNYLGPGNTDFSIEETNQSDKAAKAHDLEYNKLLQKGQNPY-IYFNHADEDFIQAT
>Protoparvovirus primate 1|YP_009507380.1/10-69 putative VP1 [Protoparvovirus primate1]
WVPPGYNYLGPFNQDFSKKPTNPSDNAARKHDLEYNKLIKQGHNPY-WNYNHADEDFIKET
>Cutavirus|YP_009508805.1/10-69 putative VP1 [Cutavirus]
WVPPGYNFLGPFNQDFNKEPTNPSDNAAKQHDLEYNKLINQGHNPY-WYYNKADEDFIKAT

```
>Tusavirus 1|YP_010796333.1/11-70 putative VP1 [Tusavirus 1]
WVPPGYNYLGPGNDLDAGEPTNKSDAAARKHDFAYSAYLKQGLDPY-WNFNKADEKFIRDT
>Fox parvovirus|YP_010797208.1/12-71 VP1 [Fox parvovirus]
WVPPGYKYLGPGNALDQGEPTNPSDAAAKEHDEAYSKYLESGKNPY-LYYNPADATFIDKT
>Megabat bufavirus|YP_009241377.1/16-75 capsid protein VP1 [Megabat bufavirus
1]
WVPPGYNYLGPGNTNFKKQPTNPSDKAARNHDLEYNKILKEGQNPY-IYFNHADEDFIKAT
>Rat bufavirus|YP_009186841.1/11-70 VP1 [Rat bufavirus SY-2015]
LTLPGYNYLGPGNTDFTKKPTNPSDEAARRHDLAYGSYIKKGHNPY-FNFNKADKSFIKET
>YP_009130651.1/11-70 capsid protein VP1 [Protoparvovirus eulipotyphla1]
LTLPGYNYLGPGNSDFTKKPTNASDAAARKHDLAYGSYLKKGHNPY-INFNNADKTFIKDT
>Minute virus of mice|NP_041244.1/12-71 hypothetical protein MMVgp3 [Minute
virus of mice]
WVPPGYKYLGPGNSLDQGEPTNPSDAAAKEHDEAYDQYIKSGKNPY-LYFSAADQRFIDQT
> Newlavirus|YP_010805432.1/11-70 VP1 [Newlavirus]
WVPPGYKYLGPGNSLDKGTPSNPSDAAAQRHDHAYSAYQAAGFNPY-FYHNQADENFIRDT
```
