# Supplementary figures and images for: Parvovirus B19 and Human Parvovirus 4 Encode Similar Proteins in a Reading Frame Overlapping the VP1 Capsid Gene
Source: Viruses. 2024 Jan 26;16(2):191. doi: 10.3390/v16020191 (PMC10891878; doi:10.3390/v16020191)

# S1

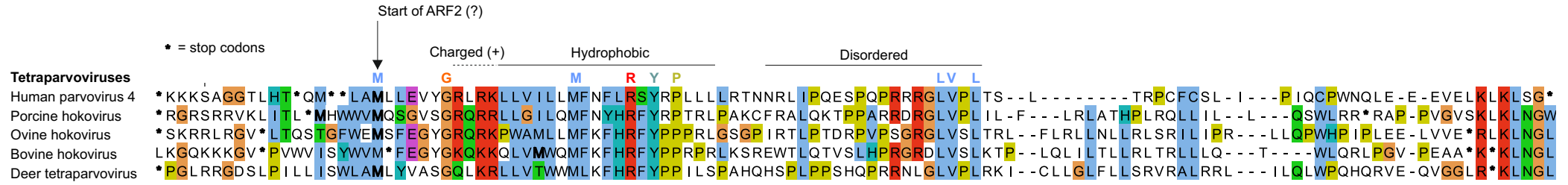

Supplement: Supplementary file 1 [file viruses-16-00191-s001.zip › S1 Fig_ARF2 tetraparvo and surrounding region.pdf]

Z protein

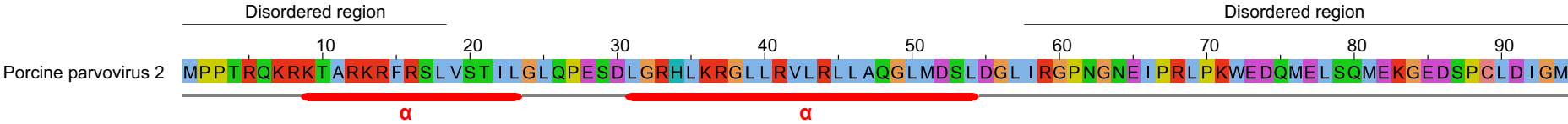

Supplement: Supplementary file 1 [file viruses-16-00191-s001.zip › S2 Fig_Z protein Porcine parvovirus 2.pdf]
